# B20 Weyl Semimetal CoSi Film Fabricated by Flash-Lamp Annealing

**DOI:** 10.1021/acsami.3c05634

**Published:** 2023-06-16

**Authors:** Zichao Li, Ye Yuan, René Hübner, Lars Rebohle, Yan Zhou, Manfred Helm, Kornelius Nielsch, Slawomir Prucnal, Shengqiang Zhou

**Affiliations:** † Helmholtz-Zentrum Dresden-Rossendorf, Institute of Ion Beam Physics and Materials Research, Bautzner Landstrasse 400, 01328 Dresden, Germany; ‡ Songshan Lake Materials Laboratory, Dongguan, Guangdong 523808, People’s Republic of China; § Institute of Materials Science, Technische Universität Dresden, 01069 Dresden, Germany; ∥ School of Science and Engineering, Chinese University of Hong Kong, Shenzhen, Guangdong 518172, China; ⊥ Institute of Applied Physics, Technische Universität Dresden, 01062 Dresden, Germany; # Institute for Metallic Materials, IFW Dresden, Dresden 01069, Germany

**Keywords:** Weyl semimetal, thin film, solid-phase epitaxy, CoSi, flash-lamp annealing

## Abstract

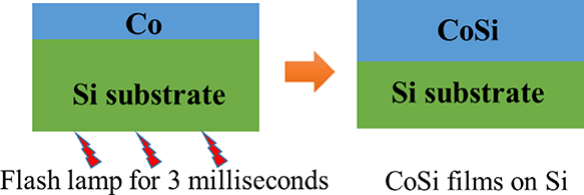

B20-CoSi is a newly discovered Weyl semimetal that crystallizes into a noncentrosymmetric crystal structure. However, the investigation of B20-CoSi has so far been focused on bulk materials, whereas the growth of thin films on technology-relevant substrates is a prerequisite for most practical applications. In this study, we have used millisecond-range flash-lamp annealing, a nonequilibrium solid-state reaction, to grow B20-CoSi thin films. By optimizing the annealing parameters, we were able to obtain thin films with a pure B20-CoSi phase. The magnetic and transport measurements indicate the appearance of the charge density wave and chiral anomaly. Our work presents a promising method for preparing thin films of most binary B20 transition-metal silicides, which are candidates for topological Weyl semimetals.

## 1 Introduction

Cobalt monosilicide (CoSi) with a B20 crystal structure is a newly discovered Weyl semimetal due to the breaking of spatial inversion symmetry.^1−3^ In particular, B20-CoSi hosts two types of chiral topological fermions, a spin-1 chiral fermion and a double Weyl fermion in the center and corner of the Brillouin zone, respectively.^4−6^ Acting as a novel platform to study topological phenomena, bulk B20-CoSi was confirmed to exhibit very long Fermi arcs^7,8^ and chiral anomaly. The material was fabricated either by the chemical vapor transport method or by the floating zone method at high temperatures.^9−11^ One common feature in Co-Si compounds is that Co_2_Si or CoSi_2_ always coexists with B20-CoSi, and the Co-Si system with a ratio of Co and Si of around 1:1 is often used as a classical example to study eutectic growth.^12−14^ Co_2_Si is a strongly ferromagnetic material in thin films or nanoparticles, while it is a weakly magnetic material in the bulk form.^15,16^ CoSi_2_ has been reported to be a superconductor with a critical temperature of around 1.5 K and has been used as an ohmic contact material for Si microelectronics.^17,18^ In the phase diagram of the Co-Si system, Co_2_Si and CoSi_2_ are two close neighbors of the B20-CoSi phase: above around 1000 °C, the formation range of CoSi is broad, while it is very narrow in the low-temperature region. Therefore, most of the published papers are based on bulk CoSi grown at high temperatures.

It is known that thin films grown on substrates of technological relevance are appreciated for many practical applications.^19−21^ However, based on our current knowledge, there are only few studies about Weyl semimetal B20-CoSi films. In early reports about the growth of Co-silicide thin films, it was found that Co_2_Si and CoSi_2_ always coexist with B20-CoSi.^22,23^ Therefore, separating the Co-Si compounds is challenging but is necessary for understanding the topological properties of CoSi films. Tang et al. have first prepared polycrystalline CoSi films on Al_2_O_3_ by magnetron sputtering. They studied the spin Hall effect by using CoSi/CoFeB/MgO heterostructures.^24^ We have prepared single-phase B20-MnSi films by flash-lamp annealing thanks to their fast heating and cooling rates.^25,26^ By controlling the heating rates and the annealing temperature, different phases can be separated. Thus, this method probably offers a possibility to fabricate pure B20-CoSi films.

Here, we report the preparation of B20-CoSi films by flash-lamp annealing, which induces a fast reaction between the predeposited Co metal film and the Si substrate. By controlling the annealing temperature, B20-CoSi films without parasitic phases are successfully fabricated, as proven by X-ray diffraction, Raman scattering, and magnetization measurements. Transmission electron microscopy analysis also confirms the formation of stoichiometric B20-CoSi. Although the B20-CoSi film is polycrystalline, it shows signatures of the charge density wave and room-temperature chiral anomaly. Our preparation method can be a general phase-selective approach for the exploration of new Weyl semimetal films.

## 2 Experimental Section

### 2.1 Film Fabrication

In order to fabricate CoSi films, 25 nm Co films were first deposited on Si(100) wafers by DC magnetron sputtering. Then, the Co films were covered by 20 nm amorphous Si to avoid oxidation. Afterward, flash-lamp annealing (FLA) was employed to realize a fast solid-state reaction between Co and Si. During the FLA process, these samples were heated up by 12 Xe lamps in a continuous N_2_ flow.^26^ Samples were annealed from the rear side with different energy densities. With a 3 ms pulse duration, the heating and cooling rates were estimated to be in the magnitude of 10^5^ and 10^2^ ks^–1^, respectively. Such high heating/cooling rates will allow the control over the parasitic growth of Co_2_Si and CoSi_2_ in B20-type CoSi. By changing the flash-lamp energy (and therefore the peak temperature), we can selectively prepare films with the pure phases of CoSi and Co_2_Si or their mixture. More details about this method were reported in ref (26). The terms 3.8R, 4.0R, and 4.2R represent the samples annealed from the rear side by flash pulses of 3.8, 4.0, and 4.2 kV (the voltage applied to the capacitor of the flash lamps), corresponding to energy densities of 46, 50.5, and 55.5 J/cm^2^, respectively.

### 2.2 Structural Characterization

Grazing-incidence X-ray diffraction (GIXRD) was employed to analyze the crystalline phases in the obtained films. GIXRD was done on a Bruker D8 Advance diffractometer with a Cu-target source (Cu Kα, 40 keV, 40 mA) at a grazing incidence angle of 5°. Micro-Raman experiments were done using a Horiba micro-Raman system with an excitation wavelength of 532 nm and a spot size of around 1 μm, and the signal was recorded with a liquid-nitrogen-cooled silicon CCD camera. Cross-sectional bright-field and high-resolution TEM images were recorded with an image-C_s_-corrected Titan 80–300 microscope (FEI) operated at an accelerating voltage of 300 kV. High-angle annular dark-field scanning transmission electron microscopy (HAADF-STEM) imaging and spectrum imaging analysis based on energy-dispersive X-ray spectroscopy (EDXS) were performed with a Talos F200X microscope (FEI) operated at 200 kV to obtain the elemental composition.

### 2.3 Magnetic and Electrical Properties

The magnetic properties of the films were measured by a superconducting quantum interference device equipped with a vibrating sample magnetometer (SQUID-VSM) with the field parallel (in-plane) to the films. The transport properties of the CoSi films were investigated by a Lake Shore Hall measurement system. Magnetic-field-dependent resistance was measured between 5 and 300 K using the van der Pauw (out-of-plane) or 4-point-probe (in-plane) geometry on samples with a size of around 5 × 5 mm^2^. Ag paste was used to contact the sample. The magnetic field was applied parallel to the sample surface plane (in-plane) or normal to the sample surface plane (out-of-plane). To observe the chiral anomaly, an in-plane magnetic field was applied parallel or vertical to the electrical field.

## 3 Results and Discussion

### 3.1 The Phase Formation and Microstructure

**Figure 1:**
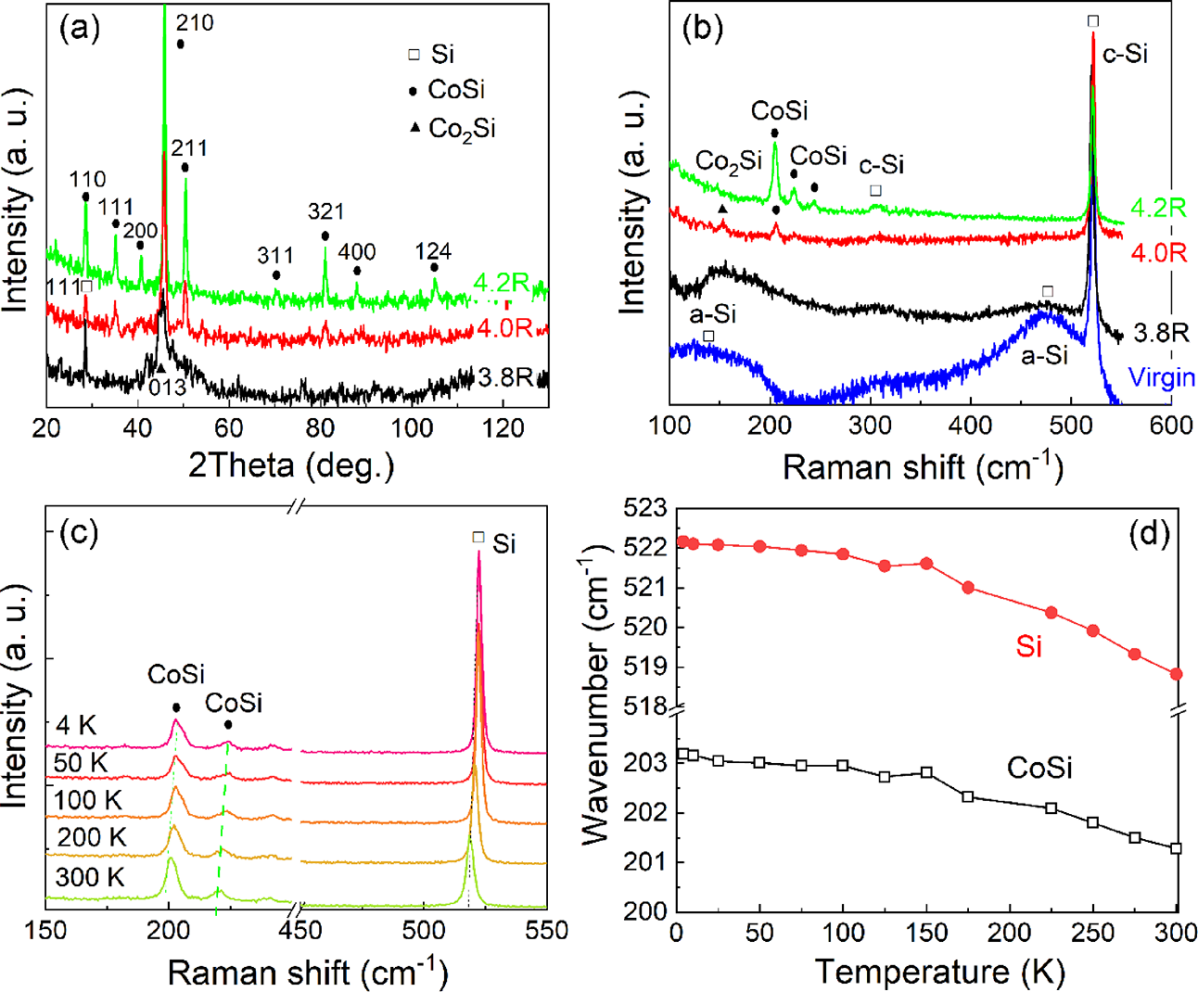
(a) GIXRD patterns and (b) Raman spectra of the annealed films on Si(100) by FLA. Sample 4.2R, annealed at the highest temperature, shows only the B20-CoSi phase. (c) Raman spectra of the annealed sample 4.2R measured at different temperatures. (d) Raman peak position as a function of the measurement temperature for CoSi (4.2R) and Si.

X-ray diffraction is a fast and nondestructive method that can be used to monitor the structural evolution of thin films depending on the preparation and annealing parameters.^27^Figure 1a displays the GIXRD patterns obtained from the samples prepared at different annealing parameters. For sample 3.8R (black, bottom curves) annealed at a low temperature, there is a broad Bragg peak present at around 45.3°, which may be from unreacted Co(111) or the newly formed Co_2_Si(013) or CoSi(210). The diffusion is limited by the low annealing temperature, which results in unreacted residual metallic Co and a low-temperature Co-rich phase, like Co_2_Si. Another peak at around 28.3° is from Si(111), which originates from the partially crystallized Si capping layer. Upon increasing the annealing energy to 50.5 J/cm^2^, the Bragg peak at 45.3° becomes narrower, and some new peaks appear, indicating the improvement of crystal quality and the formation of new phases. At this annealing energy, the peak at 28.3° becomes broader and shifts to a higher scattering angle of 28.4°, which is very probably the contribution of the new phase CoSi(110). Upon further increasing the annealing energy, the interdiffusion between Co and Si gets enhanced, and the Si capping layer also reacts with these diffused Co atoms. Sample 4.2R shows more pronounced diffraction peaks, which are consistent with B20-CoSi (PDF card no. 01-081-0484).^28,29^ Thus, by controlling the annealing temperature, we successfully prepared single-phase CoSi films.

Raman spectroscopy is another useful technique for phase identification with a lateral resolution down to the (sub-)micrometer scale. To cross-check the phase formation of our samples 3.8R, 4.0R, and 4.2R, we measured 7 different positions for each sample. In general, different positions show similar results, proving the lateral homogeneity of our films. As shown in Figure 1b, the virgin sample has two broad peaks arising from the amorphous Si (a-Si) capping layer and one sharp peak at around 520.5 cm^–1^ caused by the transverse/longitudinal optical (TO/LO) phonon mode of the crystalline Si substrate (c-Si).^30^ For sample 3.8R, these two broad peaks get much weaker, indicating the beginning of the recrystallization of the amorphous Si capping layer. For sample 4.0R, one peak at around 150 cm^–1^ and another peak at around 200 cm^–1^ appear, corresponding to Co_2_Si and CoSi,^31^ respectively. It means that sample 4.0R contains two phases. With further increasing the annealing temperature, the Raman peak from Co_2_Si vanishes, and the CoSi peaks become stronger, indicating a pure CoSi phase in sample 4.2R. To confirm the preparation reproducibility, more samples are shown in the Supporting Information (Figures S1–S3).

As shown in Figure 1c, the Raman peak of B20-CoSi and Si for sample 4.2R can be detected from 4 to 300 K. With increasing the temperature, all Raman peaks for CoSi and Si shift to lower wavenumbers. The peak positions as a function of temperature are plotted in Figure 1d. The redshift with increasing temperature is well-understood as the thermal expansion. The tendency of the CoSi Raman shift is similar to Si, indicating that the thermal expansion is also the main reason for the redshift of the CoSi Raman peaks. It also indicates that there is no visible transition temperature from 4 to 300 K for CoSi films.

**Figure 2:**
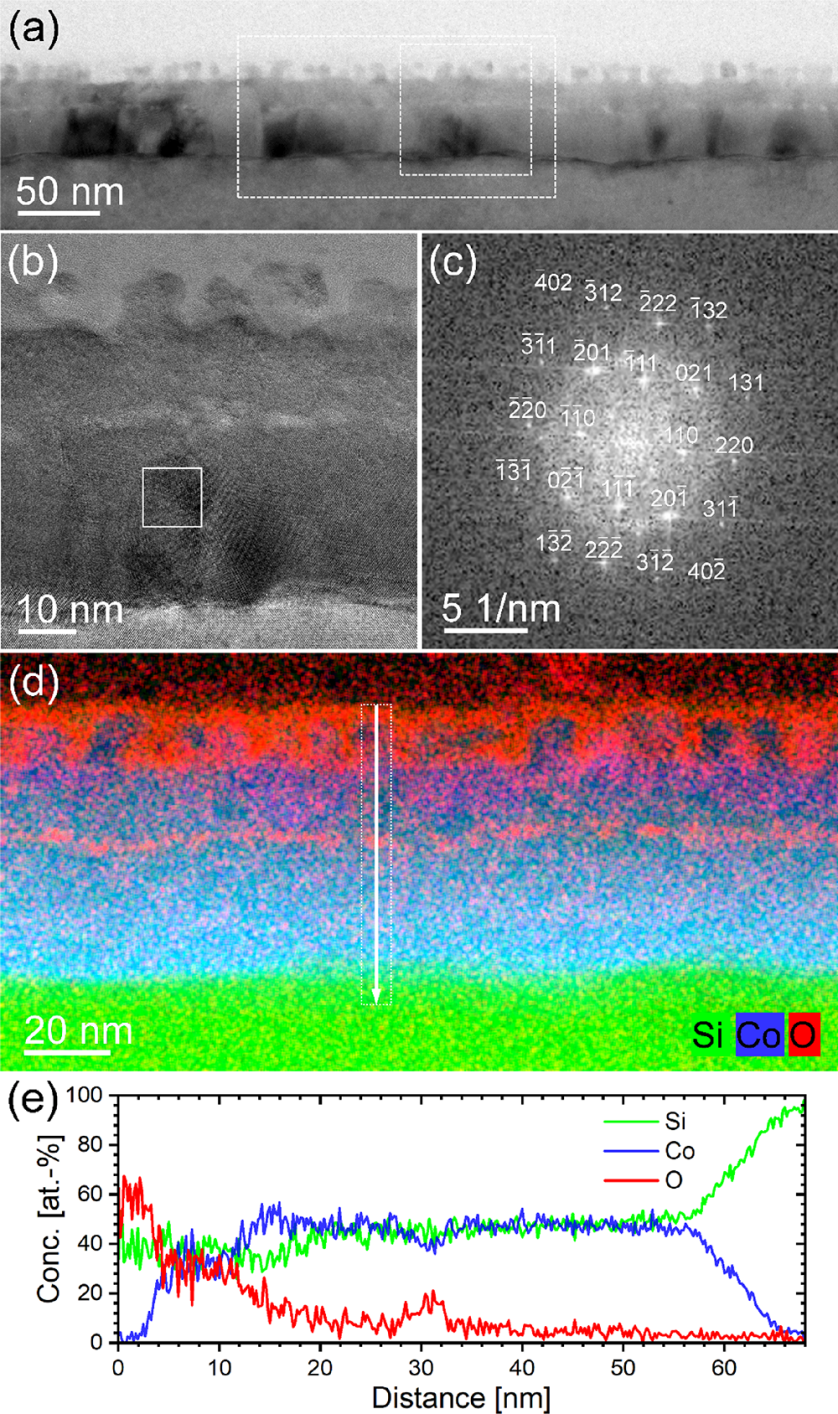
(a) Cross-sectional bright-field TEM image of the FLA-treated sample 4.2R. (b) High-resolution TEM image of the area marked with a dashed white square in panel (a). (c) Fast Fourier transform of the region marked with a white square in panel (b) and indexed based on a CoSi [11̅2] zone axis pattern. (d) Superimposed EDXS-based elemental distributions (blue: cobalt, green: silicon, and red: oxygen) for the area marked with a dashed white rectangle in panel (a), confirming the presence of a continuous B20-CoSi film. (e) Concentration–distance profile extracted from the line scan region indicated with a white arrow in panel (d).

Figure 2a shows a representative cross-sectional bright-field TEM image of sample 4.2R. On top of the Si substrate (light-gray appearance with uniform brightness), there is an about 50 nm-thick film characterized by a sharp interface to the substrate. The varying contrast of this film points to its polycrystalline nature. A light-gray bead-like line, indicating the presence of a light element (see below), divides the upper third of this layer from the lower two-thirds. The top 15 nm-thick surface region has a structured appearance. To further characterize the phase structure of the continuous polycrystalline layer in the lower two-thirds, high-resolution TEM imaging (Figure 2b) combined with fast Fourier transform analysis (Figure 2c) was performed. Taking the B20-type CoSi structure, the diffractogram in Figure 2c can be described by a [11̅2] zone axis pattern. The spatially resolved distributions of the elements Co, Si, and O were characterized by EDXS-based analysis in the scanning TEM mode and are shown in Figure 2d. In particular, Co and Si are homogeneously distributed within the lower two-thirds of the silicide layer, and the Co:Si atomic ratio is determined to be 1:1 (Figure 2e). The bead-like layer is mainly depleted in cobalt and shows a significant O signal. The 15 nm-thick structured surface region is composed of Co silicide regions between oxidized silicon (Figure 2d,e). It should be mentioned that the weak oxygen signal within the Co silicide is caused by TEM lamella side-wall oxidation during storage in air. In summary, TEM analysis confirms the formation of a continuous polycrystalline B20-type CoSi film.

### 3.2 Magnetic and Electrical Properties vs Annealing Parameters

**Figure 3:**
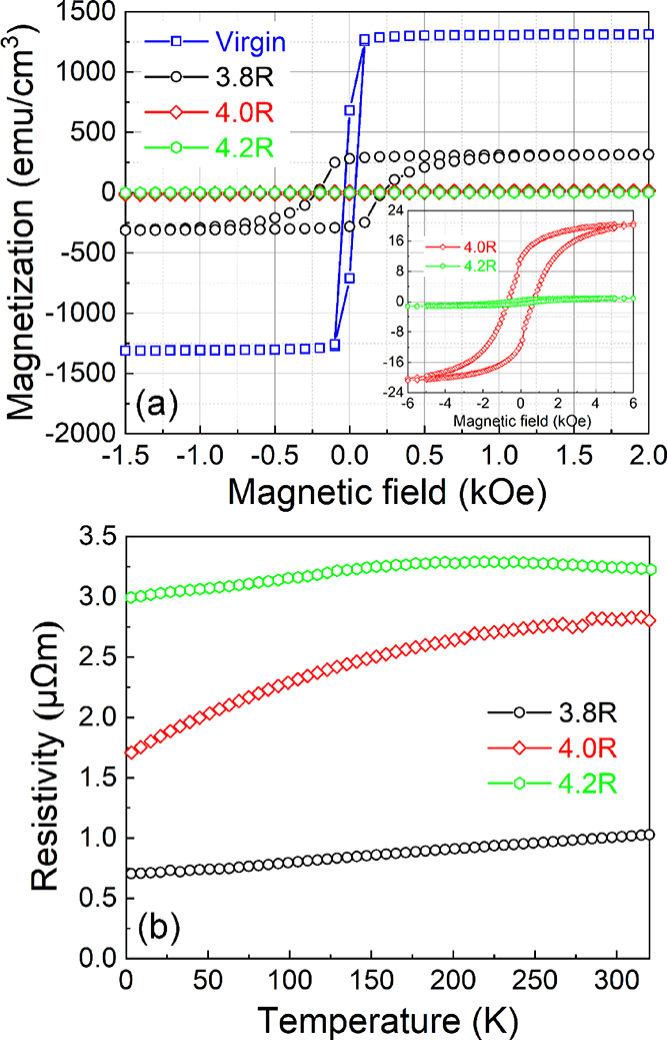
(a) In-plane *M*–*H* curves measured at 5 K for the samples flash-annealed at various annealing parameters. The inset MH curves are for samples 4.0R and 4.2R. A further zoom-in for sample 4.2R is shown as Figure S4, in the Supporting Information. (b) Temperature-dependent resistivity of all annealed samples.

The magnetic hysteresis (MH) curves measured at 5 K for all annealed samples are shown in Figure 3. The virgin sample has a strong saturation magnetization of around 1300 emu/cm^3^ and a small saturation field of around 100 Oe. The saturation magnetization is close to that of metallic Co.^32^ After annealing at an energy density of 46 J/cm^2^, the saturation magnetization decreases to 310 emu/cm^3^, and the saturation field increases to 1200 Oe. Though bulk Co_2_Si shows weak saturation magnetization, it is known that Co_2_Si thin films or nanowires show a strong ferromagnetic behavior.^15,16^ The saturation magnetization of sample 3.8R is close to the values in ref (16) (350 emu/cm^3^). With increasing the annealing energy to 50.5 J/cm^2^, most of the Co_2_Si grains transform into B20-CoSi, resulting in a dramatic decrease of the saturation magnetization to around 20 emu/cm^3^, since B20-CoSi is not ferromagnetic. Sample 4.2R with pure B20-CoSi has a negligible saturation magnetization, which is in good agreement with theory and experimental results for bulk CoSi.^33^ The zero-field cooling and field cooling magnetization measurements show consistent results regarding the phase transformation of all samples (see Figures S5 and S6 in the Supporting Information).

Figure 3b shows the temperature-dependent resistivity of all annealed films. The resistivity of sample 3.8R increases with increasing temperature, which is a typical metallic behavior.^34^ The resistivity of sample 4.0R also increases with temperature, indicating the mixed contribution of metallic Co_2_Si and semimetallic CoSi. With an increasing phase contribution of semimetallic CoSi, the resistivity increases. Sample 4.2R shows the highest resistivity and a clear deviation from metallic behavior, which will be discussed in detail in the next section. We also notice that at low temperatures, the residual resistivity in all samples is very high, leading to a low residual resistivity ratio. This is very probably due to the oxide impurities and the crystalline defects. Both can introduce additional scattering and increase the low-temperature resistivity. The out-of-plane magnetoresistance and the Hall resistance of the annealed samples are shown in Figure S7 in the supporting materials, which are totally different for the three samples composed of different Co silicide phases.

### 3.3 Magnetotransport Properties of the CoSi Film

**Figure 4:**
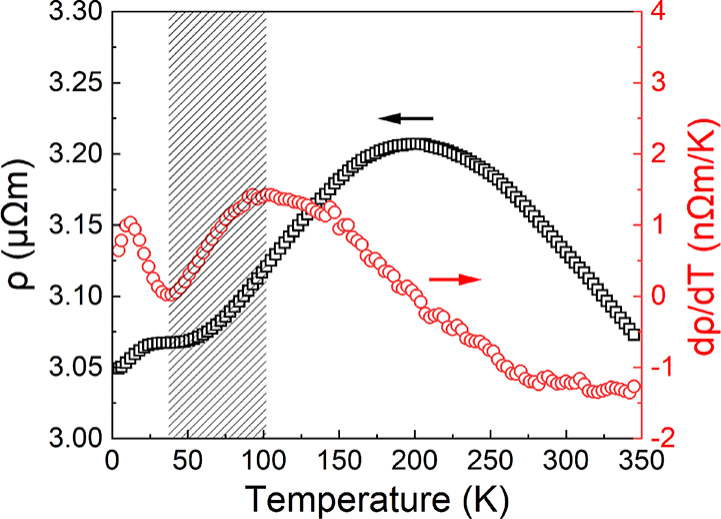
Temperature-dependent resistivity (ρ) and the calculated derivative dρ/d*T* for sample 4.2R. The shaded temperature range is the charge density wave zone.

Figure 4 shows the temperature-dependent resistivity (ρ) and the calculated derivative dρ/d*T* for sample 4.2R, which contains only B20-CoSi. Being much different from the metallic behavior, its resistivity does not show a monotonic dependence on temperature and is very close to the behavior of bulk CoSi.^34^ At around 40 K, we observe a change in the slope of the resistivity, which is more prominent in the derivative of the resistivity. The temperature coefficient of resistivity shows a broad peak at around 100 K, then decreases from 1.45 to 0.19 nΩ m K^–1^ at 40 K. Below 40 K, it starts to increase again up to 1.04 nΩ m K^–1^ at 12 K. This phenomenon is often attributed to the charge density wave (CDW) or spin density wave (SDW), but it is difficult to distinguish between CDW and SDW only by the resistivity.^35,36^ Comparing our magnetization results (Figure 3a) and the temperature-dependent magnetization (Figure S6) shown in the supporting materials, we assume that this resistance anomaly is very probably due to CDW.

**Figure 5:**
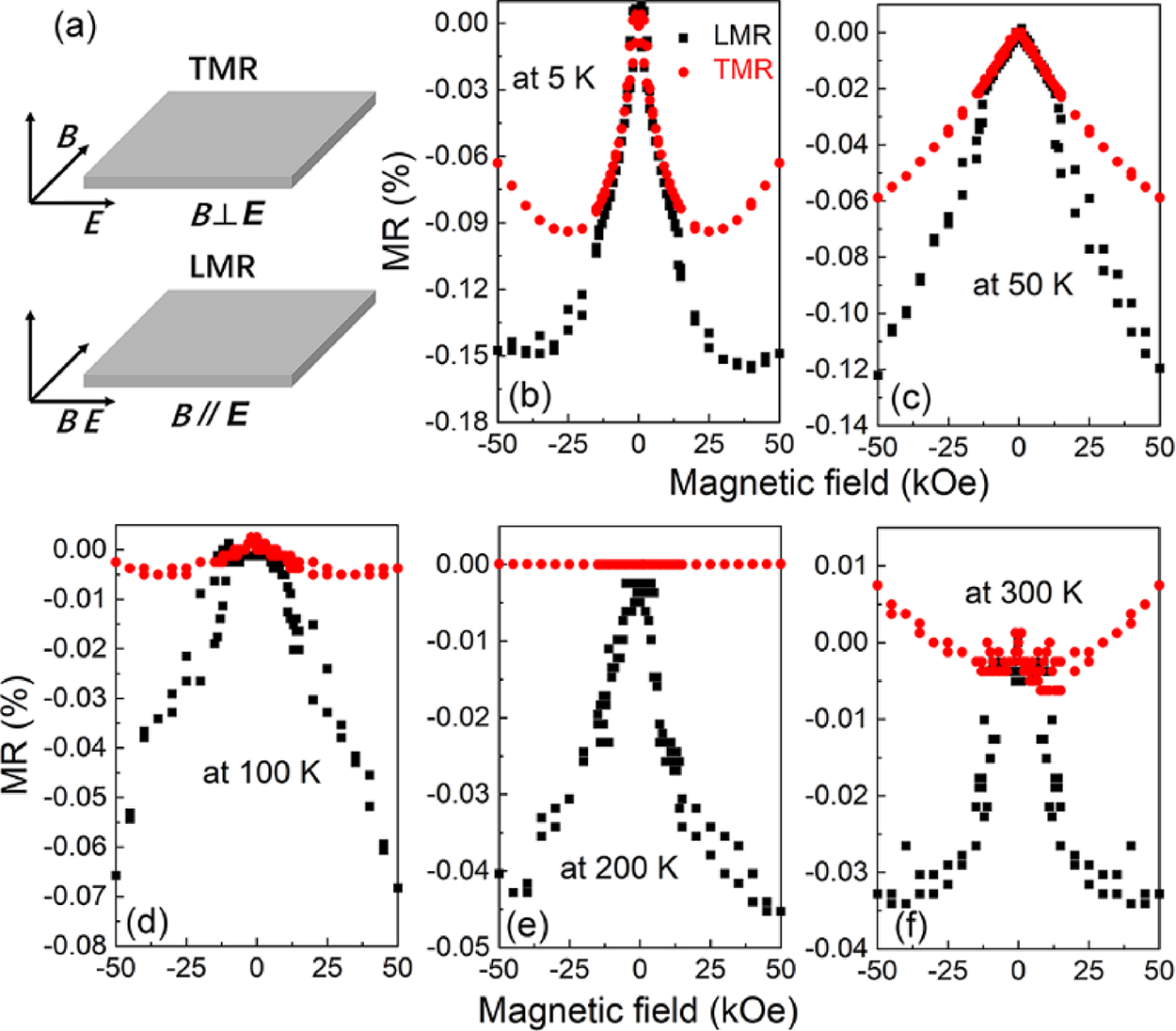
(a) Schematic for the parallel and perpendicular configuration of in-plane magnetoresistance. (b–f). LMR and TMR measurements at different temperatures.

To check the chiral anomaly in the CoSi film, we investigated the magnetoresistance (MR),  (*R*_H_ is the resistance under magnetic fields, *R*_0_ is the resistance at zero field), for different field orientations. We applied the magnetic field (B) in-plane, being perpendicular (transverse magnetoresistance: TMR) or parallel (longitudinal magnetoresistance: LMR) to the electrical field (E) (schematics shown in Figure 5a). For many topological materials like Dirac or Weyl semimetals with chiral anomaly, negative LMR and positive TMR are observed.^37−39^ The chiral anomaly is a positive correction to the magnetoconductance when the magnetic field is parallel to the electrical (LMR) since the chiral symmetry of the Weyl fermions is broken. Thus, this effect does not appear when the magnetic field is perpendicular to the electrical field (TMR).^34^ Our results at high temperatures (200 and 300 K) shown in Figure 5e,f are in agreement with these observations. At lower temperatures (Figure 5b–d), LMR is also much bigger than TMR, indicating the influence of the chiral anomaly on our sample. At low temperatures, the magnetotransport is influenced by many other effects, like the weak antilocalization/weak localization and the appearance of CDW. In ref (34), the authors tried to decouple different effects in single-crystalline CoSi microribbons. For our thin films, a detailed analysis of the magnetotransport could be possible after properly optimizing the sample growth, *i.e.*, by removing the top oxidized layer.

## 4 Conclusions

In summary, in this work, we have succeeded in preparing B20-CoSi films on Si by nonequilibrium flash-lamp annealing. Comprehensive structural characterizations point to the importance of annealing energy density to eliminate the parasitic Co_2_Si phase. The magnetic and magnetotransport properties indicate the coexistence of the charge density wave and chiral anomaly in our CoSi films. For a better understanding of the CoSi films, it is required to optimize their preparation to obtain phase-pure, epitaxial films. The nonequilibrium solid-state reaction via millisecond-range flash-lamp annealing can be a versatile approach to prepare binary transition-metal silicides and germanides.

## Supporting Information Available

Additional measurements for the structural, magnetic, and magnetotransport properties (PDF)
