# Supplementary material for: B20 Weyl Semimetal CoSi Film Fabricated by Flash-Lamp Annealing
Source: ACS Appl Mater Interfaces. 2023 Jun 16;15(25):30517–23. doi: 10.1021/acsami.3c05634 (PMC10316320; doi:10.1021/acsami.3c05634)
Supplement: Supplementary file 1 — am3c05634_si_001.pdf [file am3c05634_si_001.pdf]

Supporting Information

B20 Weyl semimetal CoSi film fabricated by flash-lamp annealing

Zichao Li[1, 2], Ye Yuan[1, 3], René Hübner[1], Lars Rebohle[1], Yan Zhou[4], Manfred Helm[1,5], Kornelius Nielsch[2, 5, 6], Slawomir Prucnal[1], Shengqiang Zhou[1] *

[1]Helmholtz-Zentrum Dresden-Rossendorf, Institute of Ion Beam Physics and Materials Research, Bautzner Landstrasse 400, D-01328 Dresden, Germany

[2]Institute of Materials Science, Technische Universität Dresden, 01069 Dresden, Germany

[3]Songshan Lake Materials Laboratory, Dongguan, Guangdong 523808, People's Republic of China

[4]School of Science and Engineering, Chinese University of Hong Kong, Shenzhen, Guangdong 518172, China

[5] University of Science and Technology of China, Hefei, Anhui 230026, China

[6]Institute of Applied Physics, Technische Universität Dresden, 01062 Dresden, Germany

[7]Institute for Metallic Materials, IFW-Dresden, Dresden, 01069, Germany

* Corresponding author: Shengqiang Zhou

E-mail address: s.zhou@hzdr.de

In this supplementary document, we present additional experimental results which are supporting the manuscript.

The sample notation is as follows:

3.8R: low processing temperature

4.0R: middle processing temperature

4.2R: high processing temperature

4.3R: highest processing temperature

1. Additional samples

To prove the reproducibility, we have prepared more samples using similar flash-lamp annealing parameters, whereby the virgin (un-annealed) Co/Si samples were from the same wafer. Two samples were prepared with low annealing energy (3.8R and 4.0R), as shown in Figure S1. Basically, these two samples are similar as in the main text. CoSi starts to form in sample 4.0R. For 4.2R, two more samples were produced with the same annealing energy, as shown in Figure S1, S2. CoSi is the dominant phase in these two samples. One sample was prepared with an even higher annealing energy (4.3R). From the Raman result (Figure S2), sample 4.3R also shows clear CoSi phase, however, in the XRD pattern (Figure S3), there is one unidentified peak, which is probably from a new phase due to the higher annealing energy.

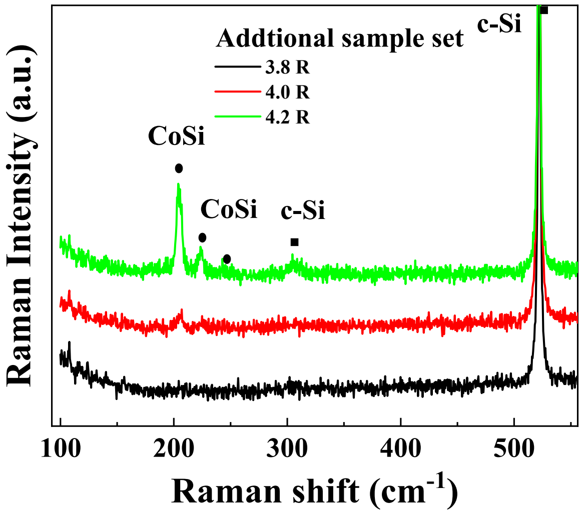

Figure S1: Raman spectra of an additional set of samples on Si(100) prepared by FLA with the annealing parameters indicated in the figure (3.8R, 4.0R, 4.2R).

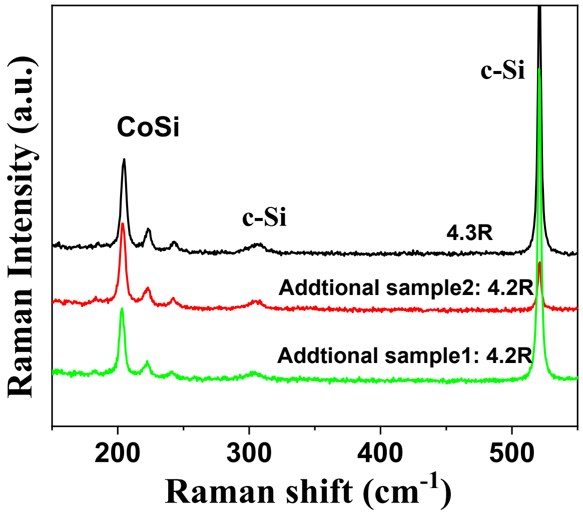

Figure S2: Raman spectra of an additional set of samples on Si(100) prepared by FLA with the annealing parameters indicated in the figure (4.2R, 4.2R, 4.3R).

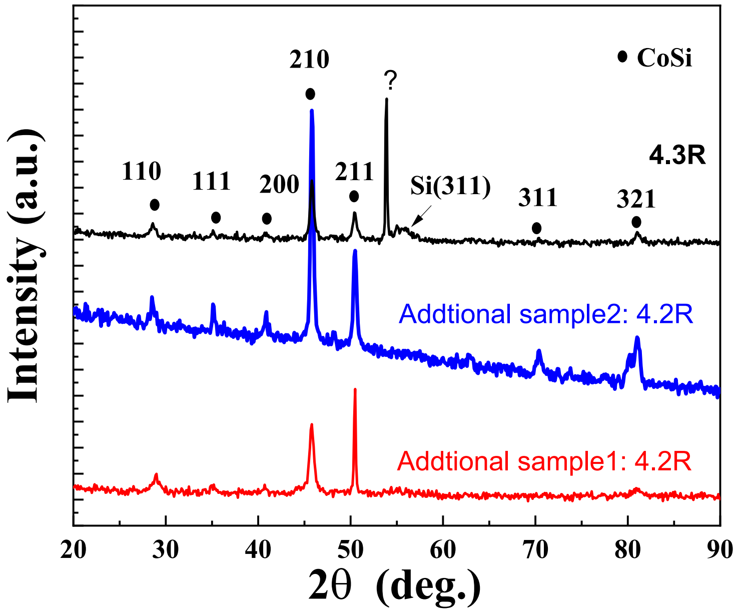

Figure S3. GIXRD patterns for additional samples on Si(100) prepared by FLA with the annealing parameters indicated in the figure. For sample 4.3R, there is one peak around 53.9° which cannot be identified at this moment. We assume they are probably from other phase due to the high temperature processing.

2. Magnetic properties

Figure S4 shows the zoom-in of the magnetic hysteresis at 5 K for the samples 4.0R and 4.2R. Sample 4.2R shows a weak magnetic hysteresis with the saturation magnetization of 1.2 emu/cm^3, 3 orders of magnitude smaller than that of ferromagnetic Co. The small magnetization could be due to some residual Co_2Si nanostructures.

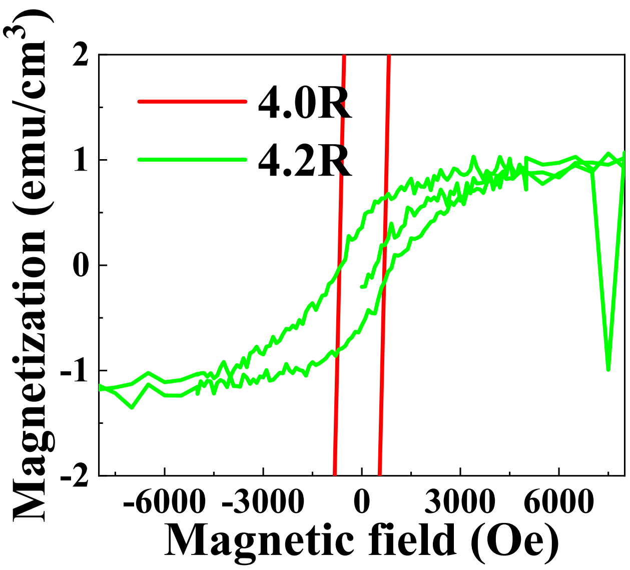

Figure S4: Zoom-in of the magnetic hysteresis at 5 K for samples 4.0R and 4.2R.

Figure S5 shows the zero field cooling (ZFC) and field cooling (FC) curves of the annealed samples 3.8R, 4.0R, and 4.2R at 50 Oe. For sample 3.8R comprising the Co_2Si phase, the ZFC and FC curves show a large magnetization compared with the other two samples and have an obvious bifurcation until 350 K, indicating the transition temperature is higher than 350 K. For sample 4.2R with single-phase B20-CoSi, the ZFC and FC curves overlap with each other with negligible magnetization, in agreement with other reports for B20-CoSi. Sample 4.0R with the phase mixture shows a decreased magnetization and a bifurcation behavior due to the superimposed magnetization of Co_2Si and CoSi.

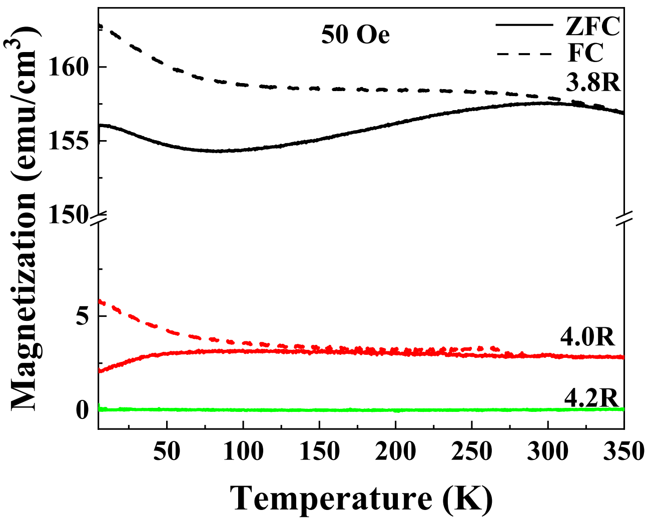

Figure S5. Zero field cooling (ZFC) and field cooling (FC) magnetization curves measured under 50 Oe for the samples annealed at different energy densities. Sample 3.8R shows a higher magnetization and transition temperature larger than 350 K. For sample 4.0R, with increasing the amount of B20-CoSi, the magnetization decreases. For sample 4.2R, the magnetization is negligible.

For further investigation of a possible charge density wave (CDW) or spin density wave (SDW) phase, we characterized the temperature-dependent magnetization of the sample 4.2R (Figure S6 (a)) at different field. The contribution of the diamagnetic Si substrate has been subtracted. At low temperature, a weak paramagnetic-like contribution also shows up: with increasing the temperature, the magnetization decreases quickly at low temperature and stays constant at high temperature. In ref. 1, the SDW phase in $Ca_3Co_4O_9$ was shown via magnetization characterization. The authors observed an anomaly in the temperature-dependent magnetization. We did not observe such anomaly, as shown by dM/dT in Figure S6 (b). Thus, we interpret the feature in the temperature-dependent resistivity (Figure 4 in the main text) as the formation of a CDW phase.

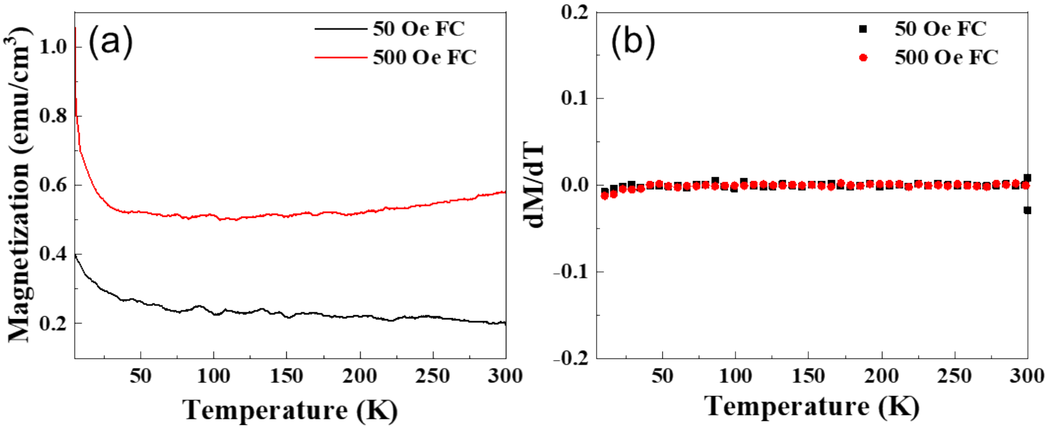

Figure S6. (a) Field cooling (FC) magnetization curves measured under 50 and 500 Oe for sample 4.2R. (b) dM/dT obtained from (a). Within the error, the curves are nearly constant without peaks in the whole temperature range, indicating that the behavior of the temperature-dependent resistivity is from CDW and not from SDW.

2. Magneto-transport properties

The magnetoresistance (MR), $MR = \frac{R_H - R_0}{R_0} \times 100\%$ (R_H: resistance under magnetic fields, R_0: resistance at zero field) and the Hall resistance of the annealed samples measured in van der Pauw geometry are shown in Figure S7. For sample 3.8R, the MR curves from 5 to 100 K show a quick saturation as the applied field exceeds 2 T and a negative MR was observed at all temperatures, like for most of the ferromagnetic materials. These MR features are similar to the reports of Co_2Si nanobelts and nanowires.[2] Due to the strong magnetization of sample 3.8R, a pronounced anomalous Hall effect is detected and shown in Figure S7 (d). As shown in Figure S7 (b) and (e), sample 4.0R behaves like a CoSi phase (Figure S7 (e)) due to the higher resistivity of CoSi compared to Co_2Si. This sample shows anomalous Hall effect (due to the Co_2Si phase) at low temperature and ordinary Hall effect at higher temperature. It is known that sample 4.2R with pure CoSi is not ferromagnetic (shown in Figure 2 (a)), in agreement with bulk CoSi measurements.[3] As shown in Figure 75 (f), there is no anomalous Hall effect but only an ordinary linear Hall effect with negative slope, indicating an n-type behavior consistent with reported data for CoSi.[4] At room temperature, the carrier concentration is around 5.6×10^{21} /cm^3

and the mobility is 3.7 cm^2/(Vs). The carrier concentration is apparently lower than that of normal metals, and the mobility is also higher.

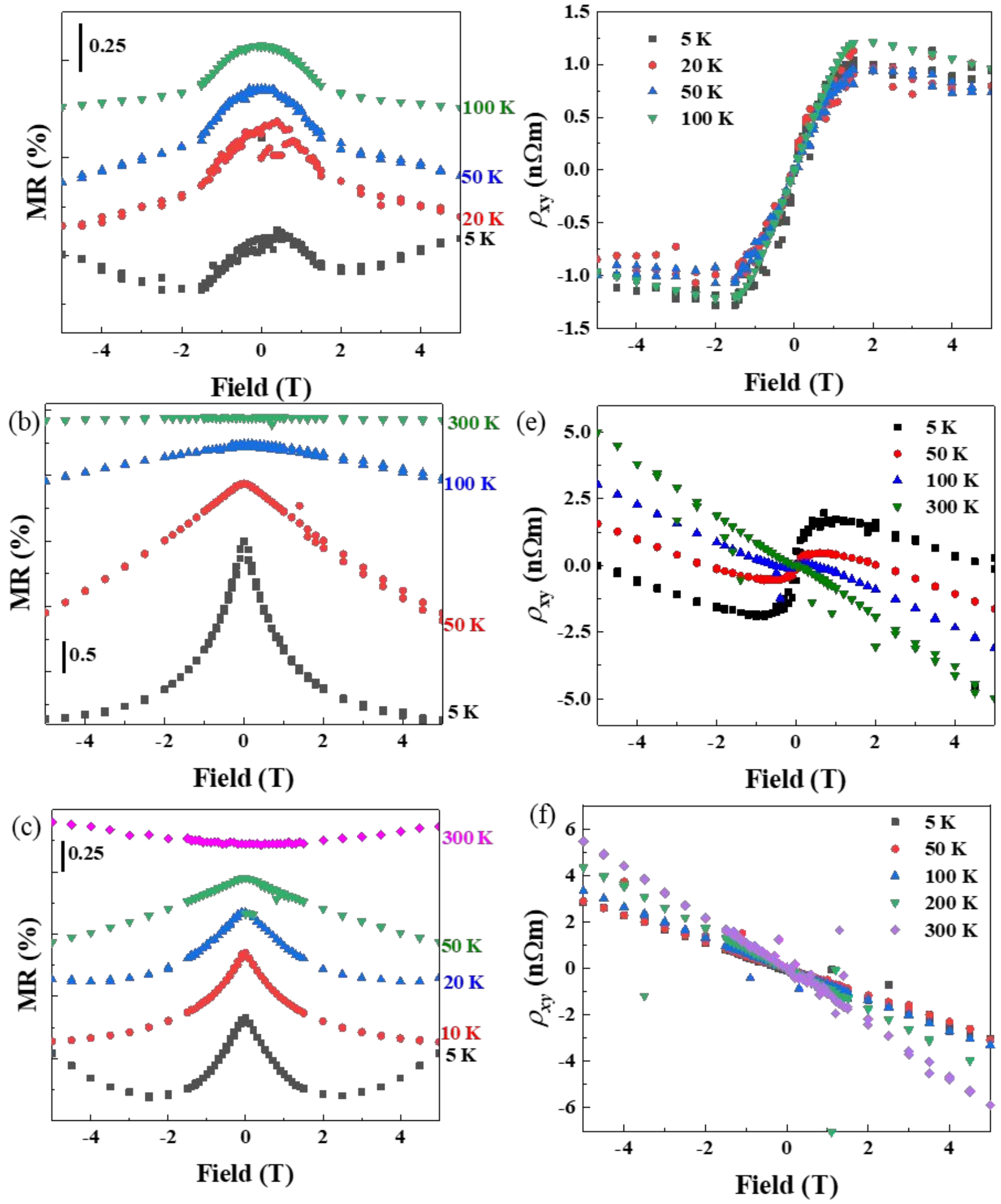

Figure S7. Out-of-plane magnetoresistance for sample 3.8R (a), 4.0R (b), and 4.2R (c). Hall effect for sample 3.8R (d), 4.0R (e), and 4.2R (f). The magnetic field was applied perpendicular to the sample surface plane. Sample 3.8R shows a totally different magnetoresistance behavior compared to the other two samples and a pronounced anomalous Hall effect. For sample 4.0R, the magnetoresistance behaves like CoSi and the anomalous Hall effect due to ferromagnetic Co_2Si becomes less pronounced compared with sample 3.8R.

References

[1] Sugiyama, J.; Xia, C.; Tani, T. Anisotropic Magnetic Properties of Ca3Co4O9: Evidence for a Spin-Density-Wave Transition at 27 K. Phys. Rev. B 2003, 67, 104410.

[2] Qu, Y.; Bai, J. W.; Liao, L.; Cheng, R.; Lin, Y. C.; Huang, Y.; Guo, T.; Duan, X. F. Synthesis and Electric Properties of Dicobalt Silicide Nanobelts. Chem. Commun. 2011, 47, 1255.

[3] Stishov, S. M.; Petrova, A. E.; Sidorov, V. A.; Menzel, D. Self-Doping Effects in Cobalt Silicide CoSi: Electrical, Magnetic, Elastic, and Thermodynamic Properties. Phys. Rev. B 2012, 86, 064433.

[4] Pan, Z.; Zhang, L.; Wu, J. Electronic Structure and Transport Properties of Doped CoSi Single Crystal. J. Appl. Phys. 2007, 101, 033715.
